# Polymorphisms predicting phylogeny in hepatitis B virus

**DOI:** 10.1093/ve/veac116

**Published:** 2022-12-10

**Authors:** José Lourenço, Anna L McNaughton, Caitlin Pley, Uri Obolski, Sunetra Gupta, Philippa C Matthews

**Affiliations:** BioISI (Biosystems and Integrative Sciences Institute), Faculty of Sciences, University of Lisbon, Campo Grande, Lisbon 1749-016, Portugal; Population Health Science, Bristol Medical School, University of Bristo, 5 Tyndall Ave, Bristol BS81UDBS8, UK; Guy’s and St Thomas’ NHS Foundation Trust, Westminster Bridge Rd, London SE1 7EH, UK; School of Public Health, Tel Aviv University, Tel Aviv 6997801, Israel; Porter School of the Environment and Earth Sciences, Tel Aviv University, Tel Aviv 6997801, Israel; Department of Zoology, University of Oxford, Medawar Building for Pathogen Research, South Parks Road, Oxford OX1 3SY, UK; The Francis Crick Institute, 1 Midland Road, London NW1 1AT, UK; Division of Infection and Immunity, University College London, Gower Street, London WC1E 6BT, UK; Department of Infectious Diseases, University College London Hospital, 250 Euston Road, London NW1 2PG, UK; Nuffield Department of Medicine, University of Oxford, Medawar Building for Pathogen Research, South Parks Road, Oxford OX1 3SY, UK

**Keywords:** HBV, hepatitis B virus, hepadnavirus, diversity, selection, phylogeny, polymorphism, mutation, evolution, genotype, subgenotype, machine learning, covariation

## Abstract

Hepatitis B viruses (HBVs) are compact viruses with circular genomes of ∼3.2 kb in length. Four genes (*HBx, Core, Surface, and Polymerase*) generating seven products are encoded on overlapping reading frames. Ten HBV genotypes have been characterised (A–J), which may account for differences in transmission, outcomes of infection, and treatment response. However, HBV genotyping is rarely undertaken, and sequencing remains inaccessible in many settings. We set out to assess which amino acid (aa) sites in the HBV genome are most informative for determining genotype, using a machine learning approach based on random forest algorithms (RFA). We downloaded 5,496 genome-length HBV sequences from a public database, excluding recombinant sequences, regions with conserved indels, and genotypes I and J. Each gene was separately translated into aa, and the proteins concatenated into a single sequence (length 1,614 aa). Using RFA, we searched for aa sites predictive of genotype and assessed covariation among the sites with a mutual information–based method. We were able to discriminate confidently between genotypes A–H using ten aa sites. Half of these sites (5/10) sites were identified in Polymerase (Pol), of which 4/5 were in the spacer domain and one in reverse transcriptase. A further 4/10 sites were located in Surface protein and a single site in HBx. There were no informative sites in Core. Properties of the aa were generally not conserved between genotypes at informative sites. Among the highest co-varying pairs of sites, there were fifty-five pairs that included one of these ‘top ten’ sites. Overall, we have shown that RFA analysis is a powerful tool for identifying aa sites that predict the HBV lineage, with an unexpectedly high number of such sites in the spacer domain, which has conventionally been viewed as unimportant for structure or function. Our results improve ease of genotype prediction from limited regions of HBV sequences and may have future applications in understanding HBV evolution.

## Introduction

Hepatitis B virus (HBV) is the prototype virus of the *Hepadnaviridae* family, a family of small, circular viruses with partially double-stranded DNA genomes of ∼3.2 kb in length (McNaughton et al. 2019). The viral genome encodes seven proteins within four genes — *HBx*, *Core*, *Polymerase* (Pol), and *Surface* (Table 1; Supplementary Fig. S1) — together with associated regulatory elements (Simmonds 2001), arranged in a series of overlapping reading frames. This genome structure imposes constraints on selection, acting as a stabilising selective force during replication (Mizokami et al. 1997; Cento et al. 2013), and accounting for a reduced nucleotide substitution rate in overlapping regions (approximately 40 per cent lower than in the non-overlapping regions) (Paraskevis et al. 2015).

**Table 1.** Summary of HBV genes and proteins, and their roles and functions.

Gene	Protein(s)	Roles and function
*HBx (X)*	X	Small regulatory protein (154aa) Role in subversion of host restriction factors Transactivating properties that are implicated in oncogenesis (Tse et al. 2018)
*Core (C)*	Pre-core (e-antigen); core	Post-translational processing to derive capsid protein and e-antigen Roles in intracellular trafficking and stabilisation of covalently closed circular (ccc)DNA Soluble e-antigen is secreted into blood, can cross the placenta, and acts as an immune tolerogen
*Pol*	Pol	Four distinct domains: TP, spacer, RT, and RNAse H. Takes up approximately two-thirds of the genome (Clark and Hu 2015) RT and RNase H domains show homology to HIV proteins, and some HIV nucleos(t)ide inhibitors can be used for HBV treatment (Clark and Hu 2015). TP and spacer domains are unique, and no known homologues have been identified to date (Clark and Hu 2015)
*Surface (S)*	Short (S), medium (pre-S2 + S), long (pre-S1 + pre-S2 + S) surface proteins	External envelope Receptor binding domains Surface epitopes neutralised by vaccine-mediated or naturally arising antibodies Produced in excess, with a potential role as a tolerogen/immunological decoy Gene is completely overlapped by Pol, representing the longest known gene overlap of any animal virus (Pavesi 2015)

HBV DNA genomes are copied via RNA intermediates by means of an error-prone viral reverse transcriptase (RT) enzyme (Urban et al. 2010), driving an evolutionary rate that is higher than would be expected for a DNA virus with a high density of overlapping reading frames (McNaughton et al. 2019). The resulting genetic diversity is the basis for the classification of HBV into ten genotypes (gt), defined by ≥7.5 per cent nucleotide divergence (Kramvis 2014), and designated gt-A-I, along with an unusual recombinant putative gt-J (showing similarity to gt-C and gibbon *Orthohepadnavirus*) (Tatematsu et al. 2009). Genotypes are further classified into subgenotypes based on ≥4 per cent divergence (Kramvis 2014). There is a variation in the number of subgenotypes per genotype, ranging from more than ten subgenotypes in gt-C (McNaughton et al. 2020) (reflecting its status as the oldest lineage; Paraskevis et al. 2015) to just a single subtype in gt-E, -G, and -H.

To date, HBV sequencing (and genotyping) is not recommended at baseline by clinical guidelines and is not routinely undertaken to inform patient care, as there has been insufficient evidence to support its role in informing surveillance or determining treatment courses (Lampertico et al. 2017). However, as the pool of HBV sequence data expands, alongside linked clinical metadata, progressive insights are emerging into associations between sequence heterogeneity (including genotype, insertions, deletions, and polymorphisms) and different clinical phenotypes including treatment response and disease outcomes (Lampertico et al. 2017; Downs et al. 2020).

Machine learning approaches are frequently applied to omics-based data, including transcriptomics and proteomics (Acharjee et al. 2020). We set out to apply a machine learning approach based on a random forest algorithm (RFA) informed by full-length HBV sequences. Our aim was to identify genome regions through which genotype can be predicted and to develop a method for future exploration of viral diversity, covariation, and the selection pressures that determine HBV genetic population structure.

## Methods

RFAs are a type of decision tree-based analysis, providing a relatively hypothesis-free approach to interrogating complex datasets. The method has been applied widely, including in host tropism studies in influenza (Eng, Tong, and Tan 2014), to identify molecules inhibiting flaviviruses (Rajput and Kumar 2018), to analyse mutational fitness effects in picornaviruses (Mattenberger et al. 2021), and in the identification of genes related to immunogenicity and pathogenicity in *Streptococcus pneumoniae* infection (Lourenço et al. 2017; Obolski et al. 2019).

Briefly, this study included nucleotide alignments (*n* = 5,496) of HBV genotypes A–H (McNaughton et al. 2020) (Supplementary Table S1). Recombinant sequences were excluded from the analysis, as were genotypes I and J which are recombinant in origin. Each of the overlapping HBV genes was separately translated into amino acid (aa) sequences, which were then concatenated into a single sequence for each genome (total length 1,614 aa, Supplementary Fig. S1B). Residues were numbered and reported using X02763 (gt-A) as a reference sequence, as is convention in the field (McNaughton et al. 2020). We refer to residue positions of the sequence alignment used in the computational analyses using a prefix ‘p’ and refer to residues of the reference sequence without the prefix. The RFA pipeline, as detailed in the Supplementary methods and Supplementary Figs S3 and S4, was then applied to the concatenated HBV sequences, using the known genotype of each sequence as the classification variable and aa sites as predictive variables, in search of a parsimonious number of sites that maximised the prediction of sample genotype (feature selection). The method thus identifies sites that most reliably discriminate between all genotypes.

To address the impact of site covariation on feature selection, we quantified aa covariation among all pairs of sites in the HBV genome using a mutual information (MI) approach as previously applied to *Plasmodium falciparum* sequence data (Spensley et al. 2019). A full description of the methods can be found in the Supplementary material.

## Results

### HBV genotypes can be distinguished through ten aa sites

**Figure 1.**
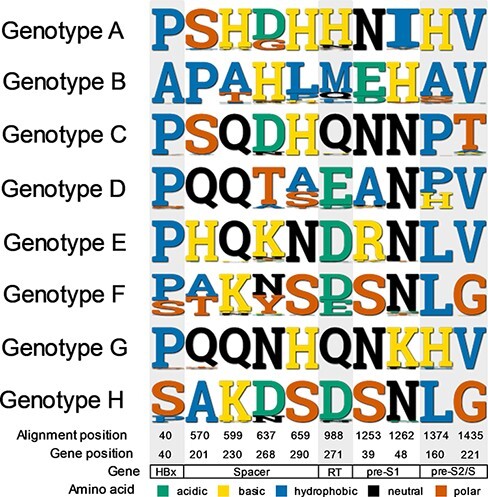
Top ten aa sites discriminating between HBV genotypes and the residues found at the sites in each genotype. Residues have been coloured according to their properties (key at bottom of the figure). Position of the sites is given in the concatenated amino construct used for analysis (Supplementary Fig. S1), and the equivalent locations in each gene are given at the foot of the figure. In Pol, residues in spacer are given assuming the first aa is at the start of the TP, and sites within the RT domain are counted separately, as is convention in the field (Supplementary Table S3).

The machine learning approach discriminated confidently between HBV gt-A-H using just ten aa sites (Fig. 1). Half of these sites (5/10) were identified in Pol, with four in the spacer region of Pol and a single site in the RT domain. A further 4/10 sites were located in the Surface protein, particularly in pre-S1 (2/4), and a single site was identified in HBx. The majority of the sites (9/10) were in overlapping regions (the single site in RT being the exception), with the pre-S1/spacer overlap accounting for 6/10 sites. None of the ten sites identified were in the Core protein.

We classified the aa sites based on chemical properties (Fig. 1). Properties were generally not conserved across the genotypes at informative sites, with the exception of HBx-40 which was almost always hydrophobic, apart from in gt-F/H. This observation suggests that there may be consistent selection pressure to maintain different chemical properties between genotypes and that the sites are not located in key regions required for host interactions, as this would typically require functional conservation.

### General location of the top genotype-informative sites

We considered the top fifty most informative sites to determine whether this changed the distribution throughout the genome compared to the top ten sites. The distribution of these sites within the genome remained comparable. In particular, aa 40 in HBx remained the only informative site in HBx, 28/50 (56 per cent) were located in Pol, with 16/28 of these sites located in the spacer domain. The Surface protein contained 21/50 sites. The majority of sites were identified in overlapping regions of the genome, with a low number of sites in the terminal protein (TP), RT, and RNAse H domains of the Pol polyprotein (Supplementary Table S2, Fig. S2).

### Sites defining genotypes

**Figure 2.**
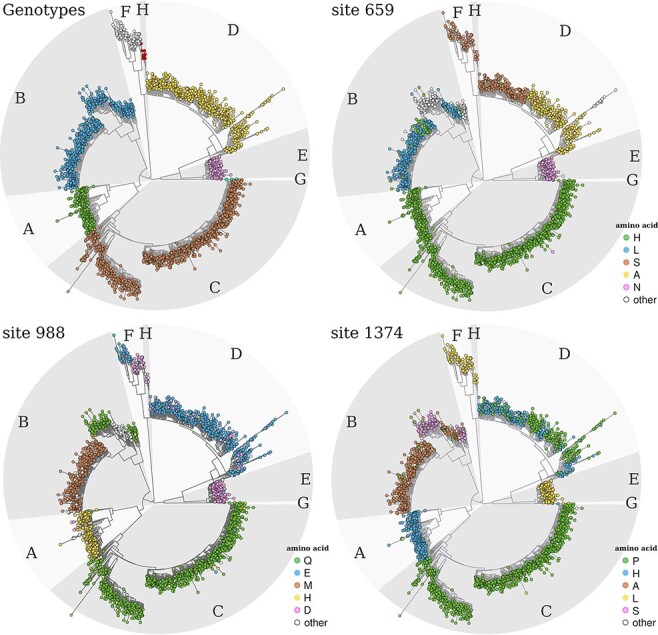
Phylogenetic trees showing overall genotype lineage and distribution of three exemplar aa sites that predict lineage. Maximum likelihood phylogenetic trees were available to download as a part of the online resource from which we obtained nucleotide sequences (McNaughton et al. 2020). The top-left tree highlights the different HBV genotypes (A to H) with capital letters and with shaded, alternating, lighter, and darker grey areas. For the trees of Sites 659, 988, and 1,374, nodes are coloured on the basis of the aa residue at each site (inner legends) using an in-house R script based on the R package ‘Analyses of Phylogenetics and Evolution’ (ape v5.4; Paradis and Schliep 2019). On each tree, only the top five most frequent amino acids are presented, with the rest under the category ‘other’. Phylogenies for the other seven top ten sites are shown in Supplementary Figs S5 and S6.

Although ten sites were sufficient to discriminate confidently between all genotypes considered, the majority of sites identified were conserved within each genotype, albeit with a few sites presenting variation at the subgenotype level. For example, gt-B sequences could be identified by a single site, 40A in HBx, with all other genotypes having 40P/S (Fig. 1, Supplementary Fig. S5). The 988H residue (271H in RT) was also key for identifying gt-A (Figs 1, 2). Other sites were polymorphic within a particular genotype, but genotype-specificity could be distinguished by the *absence* of particular residues (e.g. non-V/G at Site 1,435 (221 in surface) indicates gt-C) (Fig. 1, Supplementary Fig. S6). The close evolutionary history of gt-F and H could be seen by homology at many of the top ten sites (Fig. 1), with Site 637 (eighty-seven in spacer domain) demonstrating the clearest discrimination between gt-F (637N/Y) and gt-H (637D) (Supplementary Fig. S5). Sites 40 and 570 also showed differences in the distribution of amino acids between gt-F and gt-H.

### Sites defining subgenotypes

A number of the top ten informative sites were also highly discriminatory for some HBV subgenotypes, including p40-P/S (gt-F), p599-A/T (gt-B), p637-D/G (gt-A), and p659-A/S (gt-D) (Fig. 2, Supplementary Figs S5 and S6). Differences in the amino acids selected at some of the sites in gt-D, in particular p40-S and p1253-R (Supplementary Figs S5 and S6), also support the designation of gt-D5 as a unique subtype (McNaughton et al. 2020). These sequences cluster distantly from other gt-D sequences on a long branch and show strong geographical clustering, with all sequences isolated from India and Bangladesh.

### Co-varying sites with top ten informative sites

When using random forests, high covariation between two predictor variables can result in their importance for classification being shared and thus penalised (relative to other predictor variables). Within our pipeline, this could have resulted in the exclusion of pairs of sites that present high covariation or the exclusion of single sites that had high covariation with the top ten selected sites. To address these possibilities, we used the MI theory to quantify covariation between all pairs of aa sites across the genome. Below we provide a summary of results, but a more detailed analysis is presented in the Supplementary material. The vast majority of site pairs presented low covariation (Supplementary Fig. S7A, B). Among 361 pairs of sites with the highest covariation, 9 pairs included sites from the top 10 selected sites informative of genotype (Supplementary Table S4). When comparing the top thirty and top ten most informative sites of genotype according to the RFA approach, we show that the last twenty sites rejected did not form pairs of sites with high covariation. This reassured us that rejection of informative sites in the last steps of the RFA pipeline was not based on penalising sites that were both highly informative and highly co-varying.

Of the top 10 sites, three featured in 55/1,680 site pairs with the highest covariation (HBx Site 40 and spacer Sites p599 and p637) presenting varying degrees of covariation with a range of sites across the genome (Supplementary Table S4, Fig. S8). These other sites, although not in the top ten list of sites that discriminate genotype, could still be of biological interest. For example, we find that HBx 40 is highly co-variable with aa 227 in Pol (p596) and a series of amino acids at the start of small-HBs (p1403, p1404, p1406, and p1411; corresponding to aa 15–23), which also co-varied with Site p599, but to a lesser degree. The highest covariations were found between p599 and both HBx aa 39 (p39) and pre-S1 aa 97 (p1311), which were also found to intermediately co-vary with HBx 40. Reasons for the strong association between this site in HBx, spacer, and the start of small-HBs are unclear. While we cannot refute the null hypothesis that such associations arise by chance, under evolutionary drift, they may also point to an underlying biological influence. The majority of past work on HBx interactions has also focused on interactions with host proteins rather than considering influences on other viral proteins (Slagle and Bouchard 2018; Van Damme et al. 2021). In comparison, Site p637 (aa 268 in Pol) had the lowest degrees of covariation with other sites, but nonetheless presented a varied list of connections, showing associations with two sites in Core (aa 100 and 123; p254 and 277, respectively) and a cluster of closely located sites in Pol (p632, p633, and p636). This cluster of sites in Pol overlaps a regulatory region in the nucleotide code adjacent to a ‘CCAAT’ box, known to be the S-promoter region (Taghiabadi et al. 2019).

## Discussion

### HBV genotypes can be defined by ten key aa sites

Our analysis demonstrates that HBV genetic population structure can be determined from as few as ten aa sites across the viral proteome (Fig. 1, Supplementary Table S3). Rather than identifying sites that reliably predict a single genotype, the method identifies sites that can discriminate between multiple genotypes with a high level of certainty. Four of the top ten predictive sites were identified by previous studies as informative for HBV genotyping (Supplementary Table S3). Our approach is agnostic to nucleotide sequence, and concatenating the proteome for analysis allowed us to analyse each residue independently, avoiding difficulties in interpretation that could otherwise arise as a result of the overlapping genome structure. Our analysis further suggests that Core appears far less informative for distinguishing HBV genotypes than other genes (Supplementary Table S2). This is in keeping with a high conservation rate of >75 per cent of aa sites (Chain and Myers 2005), as expected for a highly structural capsid protein which also plays diverse roles in the viral replication cycle. HBx was also found to be a relatively uninformative region of the HBV genome, with a single site identified in the top fifty most informative sites (Supplementary Table S2). While both these proteins may contain residues that are unique to a specific genotype, there are few sites that allow reliable discrimination across genotypes.

### Informative sites are concentrated in the spacer domain

The spacer domain, which spans aa 184–348, is an intrinsically disordered protein and poorly conserved region of Pol, unique to *Hepadnaviridae*. Previous literature has shown that the spacer domain can tolerate significant deletions and insertions without a significant impact on Pol function (Bartenschlager, Junker-Niepmann, and Schaller 1990; Pley et al. 2022).

The unexpected clustering of sites that predict genotype in the spacer domain indicates that while the domain accommodates considerable plasticity, it remains highly lineage-specific. Other studies have also found that spacer mutations are relevant in distinguishing between simian HBVs (Norder et al. 1996), as well as human HBV genotypes (Zhang et al. 2010; Ingasia et al. 2020; McNaughton et al. 2020) and subgenotypes (Tallo et al. 2008; Gulube et al. 2011; Lago et al. 2014). Importantly the four top ten sites we identified in the spacer map to regions previously identified as useful for lineage distinction (Pley et al. 2022). This suggests that selection pressures may be acting to conserve genotype-specific sequences within spacer. Furthermore, it substantiates the hypothesis that spacer plays a central role in the co-evolution of the overlapping P and S genes, potentially related to selection pressure from antiviral drugs, vaccines, and the host immune response (Pley et al. 2022). In addition to encoding regions within proteins, the promoter region for the RNA transcript encoding medium- and small-HBs is present in the pre-S1/spacer overlap region. Mutation of the spacer region is therefore likely to interfere with the generation of M-/S-HBs transcripts, the biological significance of which is unclear (Pfefferkorn et al. 2018). Current models are poorly equipped to study this, suggesting that our understanding of the role of spacer may be limited by the tools used to analyse its function.

### Limitations of the methodology

The proportion of recombinant viruses circulating in the host population is yet to be robustly quantified, mostly because genotyping is still not common practice, full-length genome data are very limited and are skewed towards high viral load infections. In our analyses, recombinant sequences, combining two or more different HBV genotypes, were intentionally excluded from this analysis. As such, the short list of ten sites that can discriminate genotypes is not expected to be able to adequately classify recombinant samples (Simmonds and Midgley 2005). Since our algorithm used the aa sequences to compare isolates, synonymous mutations are not considered in the analysis. As several regions of the HBV genome contain promoter regions, such as the well-described basal core promoter of pre-core, synonymous changes in the DNA sequence would have important functional effects. Conservation of the nucleotide sequence in these regions would therefore also be key and may be lineage-specific. Site covariation and its biological relevance and influence in the selection of the top ten sites were not explored in an exhaustive manner, and they should be the topic of future research.

## Conclusions

We present the observation that HBV can be reliably genotyped using information from as few as ten sites and for the distinction of some genotypes by a single site. This is of potential practical importance if a genotype identification is desired but limited sequence data are available. Our finding that discriminatory sites are concentrated in spacer underlines the potential role and evolutionary importance of the spacer domain in the viral Pol. With the emerging importance of genotypes in HBV disease outcomes, quick approaches to genotyping from short fragments of sequence data may be of increasing practical utility, particularly in low-resource settings. Furthermore, describing the impact of selection pressure at different sites in the genome can provide insights into viral evolution and potentially contribute to mechanistic insights regarding viral persistence and pathogenesis.

## Supplementary Material

veac116_Supp

## Data Availability

Data and code are available online at Figshare: https://doi.org/10.6084/m9.figshare.21229532.v1.
