# Supplementary material for: Polymorphisms predicting phylogeny in hepatitis B virus
Source: Virus Evol. 2022 Dec 10;9(1):veac116. doi: 10.1093/ve/veac116 (PMC9825179; doi:10.1093/ve/veac116)
Supplement: veac116_Supp [file veac116_supp.zip › veac116_Supp/HBV PPP geno supp - revision 2 11_2022 clean.pdf]

Supplementary File

Polymorphisms Predicting Phylogeny in Hepatitis B Virus (HBV)

José Lourenço, Anna L McNaughton, Caitlin Pley, Uri Obolski, Sunetra Gupta, Philippa C Matthews

Supplementary Methods

Data sources

We downloaded genome-length alignments of HBV sequences genotyped A-H from HBVdb(1) in June 2018, obtaining a total of 5496 sequences. Recombinant sequences were excluded from the analysis, along with two additional isolates (FJ023674 and FR714503) identified as genotype (gt)-C recombinants. Genotypes I and J were also excluded as gt-I is recombinant in origin (comprising sequences from gt-A, -C and -G)(2) and only a single isolate of gt-J has ever been identified(3). The number of sequences in each genotype is listed in Table S1. The phylogenetic trees included in this analysis were adapted from an open-access online resource on HBV reference sequences(4).

Removing sequence indels

There are a number of highly conserved (and often considered genotype-defining) indels that have been widely described in the HBV literature (see e.g.(4)). For example, it is recognized that compared to genotype A: all other genotypes have a 6bp deletion in the core (C), at nucleotide (nt) 2354; genotypes E and G have a 3bp deletion in the pre-S1 region, at nt2861; genotypes D and J have a 33bp deletion at the start of the pre-S1 region, at nt2854; genotype G also has a 33bp insertion in the core, at nt1903. Including indel regions would result in machine learning approaches scoring high the within sites as being genotype specific. As we aimed at assessing which amino acid (aa) sites across the genome are most informative for determining genotype, we removed all indel regions present within >1% sequences to allow for exploration of how informative sites across the rest of the genome are.

Numbering of HBV genome for analysis

We followed convention in the field, using the gt-A sequence X02763 as a numbering reference and defining nucleotide (nt)1 in the middle of an EcoR1 restriction site in the P/S overlapping region(4)(Figure S1A). In order to analyse the amino acid sites in each HBV gene independently, without confounding by overlapping regions, we translated each of the genes from nucleotide into amino acid separately. The translated amino acid sequences were then

concatenated into a single sequence, comprising the X, core, polymerase and surface genes (total 1613 amino acids based on a gt-A reference, Figure S1B). Regions with conserved deletions or insertions were excluded from the analysis in all sequences. All deletions removed resulted in proteins that were still in-frame and the locations of them are described by McNaughton *et al*, 2020(4).

Random forest classification approach

Using random forests, we developed a pipeline that searches for the top M most informative amino acid sites, in terms of classifying genotypes. M can be any number of amino acid sites and the pipeline stops when that number is reached or when it can no longer guarantee to reach M. This pipeline is described in detail below, and a visualisation is included as Figure S3.

Stage 1 – reduce number of independent variables

(1) Start by running a series of RFAs with the intention of finding a set of amino acid sites that are generally highly informative and much smaller in number than the length of our sequence alignment.

(1.a) Create K training-test folds (K=5). Folds are data partitions, created by randomly sampling ~80% of the dataset for training and the remainder ~20% for testing. Sampling is done such that the number of samples of each genotype is the same between sets.

(1.b) For each pair of training-test K folds run an RFA and memorize the top N' most informative sites.

(1.c) Next, create a set of N most informative sites as the intersection of the N' top sites of each K RFA run. The set N is of minimum size N', but can be larger since each K RFA run may result in some of the N' top sites being different from the other K RFA runs.

Stage 2 – feature selection (site selection)

(2) Perform feature (site) selection among the N set, by attempting to iteratively remove the less informative sites while minimizing prediction error. First, set a minimum number of sites of interest, M (the number for which we wish to stop). Then, run the RFA with the N set of sites and order them by their resulting importance.

(2.a) Remove the site with lowest importance, resulting in a proposed new set N.

(2.b) Run the RFA with the new N set and check for an error heuristic (see below for definition).

(2.b.i) If the heuristic is not violated, then the site is permanently removed from the N set. If the size of the set N is still larger than M, the process jumps to step (a); otherwise the process stops.

(2.b.ii) If the heuristic is violated, the last removed site is returned to the N set and the site with the next lowest importance is removed. The process jumps to (b).If the removal of all sites in N is attempted consecutively without any removal, the process stops.

Within the pipeline, we took the simple heuristic of minimizing the miss rate (false negative) across genotypes. This is done by strictly not allowing the miss rate (false negative) of any genotype to be above 2.5%. Thus, in step 2.b of the pipeline, if the miss rate (false negative) of all genotypes is below 2.5%, step 2.b.i is performed, otherwise step 2.bii. In the context of the 8 classes (genotypes), the miss rate (false negative) of each can be calculated from the known versus predicted classification proportions across all samples (Table S0).

Table S0: Example of genotype prediction table

known genotype \ predicted as genotype	A	B	C	D	E	F	G	H
A	pAA	pAB	pAC	pAD	pAE	pAF	pAG	pAH
B	pBA	pBB	pBC	pBD	pBE	pBF	pBG	pBH
C	pCA	pCB	pCC	pCD	pCE	pCF	pCG	pCH
D	pDA	pDB	pDC	pDD	pDE	pDF	pDG	pDH
E	pEA	pEB	pEC	pED	pEE	pEF	pEG	pEH
F	pFA	pFB	pFC	pFD	pFE	pFF	pFG	pFH
G	pGA	pGB	pGC	pGD	pGE	pGF	pGG	pGH
H	pHA	pHB	pHC	pHD	pHE	pHF	pHG	pHH

Miss rate (false negative) of genotype i = $\text{Miss}_i = \frac{\sum_j \; pij, i \neq j}{pii + \sum_j \; pij, i \neq j}$, where pij is the proportion of samples of genotype i that were classified as j.

When using the concatenated sequences to run RFA, we use each amino-acid position as an independent variable (with the set of observed amino-acid “letters” as categorical values considered). We used K=5 (see Figure S3 and text above), node size (for classification) equal to 1, mtry (number of independent variables randomly sampled as candidates at each split) set to square-root of the total number of variables, and ntree (number of trees to grow) was set to either 200 for step 1.a or 400 for step 2.b of the pipeline (see Figure S3 and text above). The values for ntree were selected after visual inspection of no significant changes in out-of-bag error rates for higher ntree values, and opting for a small ntree number for performance reasons. The pipeline, including all RFA runs and post-analyses were performed in R (v4.1).

We used the R-packages randomForest (v4.7-1.1), caret (v6.0-90), ROCR (v1.0-11), and pROC (v1.18.0).

We use two measures of (classification) performance to present results: accuracy and multi-class area under the receiver operating characteristic curve (multi-class AUC).

Accuracy (ACC) is the proportion of samples correctly classified: $ACC_i = \frac{pii}{\sum_i \sum_j pij}$, where pij is the proportion of samples of genotype i that were classified as j. Multi-class AUC was calculated using multiple ROC curves as proposed by Hand and Till (5). Visualisation of pipeline performance is provided in Figure S4. Visualization of final out-of-bag error rates when using the selected, top 10 most informative sites for genotype classification are visualized in Figure S8.

Sites that co-vary with the top 10 sites selected by the Random Forest pipeline

When using RFA, an amino acid site's importance is a score of how important the site is to classify the genotype of a sample. If two sites are highly predictive of genotype but are also highly correlated with each other, they will present a "shared importance" score, reflecting that they hold redundant information. In the RFA pipeline we develop which seeks to perform a minimization task of selecting the shortest set of sites predictive of genotype, it is theoretically possible that: (a) sites that are informative to classify genotype but that are highly correlated may end up not being selected in the final shortest set of N sites, or (b) sites that have not been selected in the shortest set of N sites may be highly correlated with selected sites and they could thus be sites of biological interest.

To assess these two possibilities, we used Mutual Information (MI) to quantify site co-variation between every pair of sites in the sequence alignment. MI is an aggregate measure of entropy between two variables, varying between 0 and 1, with 1 representing full co-variation (every time a site changes amino acid, so does the other site). We have previously applied normalized / scaled MI to genetic sequences (full details are available in Supplementary Text of (6)). The resulting MI distribution is long tailed (Figure S7B), with the vast majority of site pairs presenting low co-variation and a small number of site pairs presenting high co-variation. We considered two thresholds of MI>0.6 and MI>0.8 to explore whether site pairs with high co-variation included sites in the top 10 list selected by the RFA pipeline. The threshold of 0.6 for intermediate-to-high MI resulted in 1680 pairs of sites (Figure S7B) that included 55 pairs of sites that included sites from the top 10 list selected by the RFA (Table S4). The threshold of 0.8 for high MI resulted in 361 pairs of sites (Figure S7B) that included 9 pairs of sites that included sites from the top 10 list selected by the RFA (Table S4 in orange).

Our RFA approach selected a short list of informative sites to predict genotype in an interactive process by reducing the list of informative sites one by one (Figure S3). To address the possibility that (a) sites that are informative to classify genotype but that are highly correlated may end up not being selected in the final shortest set of N sites, we explored the set of sites that were rejected in the intermediate RFA steps from the top 30 to the top 10 most informative sites (Table S2). The rationale was that highly informative sites in the top 30 could have been rejected to be part of the top 10 due to point (a). The 20 sites not selected for the top 10 but that existed in the top 30 were: p490, p607, p621, p624, p625, p655, p666, p688, p839, p868, p1092, p1268, p1305, p1322, p1346, p1392, p1452, p1473, p1531, and p1547. Among these, only site p1305 was featured in pairs of sites with intermediate-to-high covariation (N=19, with sites p42, p477, p495, p558, p575, p579, p580, p598, p628, p664, p745, p808, p811, p1394, p1351, p1396, p1457, p1460, p1507) or high covariation (N=4, with sites p580, p628, p1394, p1396). There were thus no top 30 informative sites that had high co-variation *with one-another* and that later were rejected to achieve the top 10 list of sites. This reassured us that rejection of informative sites in the last steps of the RFA pipeline were not based on penalizing sites that were highly informative and highly co-varying.

Of the top 10 sites, only sites p40, p599 and p637 presented high co-variation levels with other sites in the alignment (Figure S7C-E), suggesting that these other sites hold redundant information that may be of biological interest. We describe these other sites in the main text and Table S3, thus addressing point (b) above.

Supplementary Tables

Table S1: Number of full genome length HBV sequences in each genotype downloaded from HBVdb(4) and included in the analysis

HBV genotype	Number of sequences included in analysis	Number of sequences available
A	755	794
B	1430	1450
C	1805	1830
D	856	882
E	246	250
F	225	226
G	36	38
H	26	26

Table S2: List of 50 most informative sites in the concatenated HBV genome.

Number of informative sites	HBx	Core	Pol	Surface
N=10	40	n/a	570 599 637 659 988	1253 1262 1374 1435
N=20	40	n/a	570 599 624 625 637 659 666 839 988	1253 1262 1305 1322 1346 1374 1392 1435 1452 1547
N=30	40	n/a	490 570 599 607 621 624 625 637 655 659 666 688 839 868 988 1092	1253 1262 1268 1305 1322 1346 1374 1392 1435 1452 1473 1531 1547
N=40	40	n/a	452 490 570 599 607 610 621 624 625 637 639 655 659 660 666 688 771 839 868 988 1092	1253 1262 1268 1288 1305 1322 1329 1346 1374 1392 1433 1435 1444 1452 1473 1522 1531 1547
N=50	40	n/a	439 445 452 490 570 585 599 602 607 610 621 624 625 637 639 655 658 659 660 666 683 688 771 838 839 868 988 1092	1253 1262 1265 1268 1288 1305 1322 1329 1346 1374 1392 1433 1435 1444 1452 1473 1501 1502 1522 1531 1547

Table S3: Top-10 informative amino acid sites for determining HBV genotypes A-H identified using the RFA analysis. Location of the sites in the concatenated construct used for analysis, the gene and domain/open reading frame (ORF) are provided.

Position in concatenated construct	Gene	Protein aa site	Domain/ ORF	Domain/ ORF aa site	Further information
**40**	X	40	X	40	Located within 'flexible loop region' (residues 27–82); HBx site 40 has been previously identified as a site that is informative for genotyping(7)
**570**	P	201	Spacer	20	Nothing found
**599**	P	230	Spacer	49	Nothing found
**637**	P	268	Spacer	87	Nothing found

Position in concatenated construct	Gene	Protein aa site	Domain/ ORF	Domain/ ORF aa site	Further information
**659**	P	290	Spacer	109	Nothing found
**988**	P	629	RT	271	Identified in previous studies as a key discriminatory site for gt-B and gt-C(8, 9).
**1253**	S	39	S1	39	Located within the NTCP-binding region of preS1 (aa2-48)(10); Neutralising antibodies can target region(11); Identified in previous genotyping studies based on pre-S1(12)
**1262**	S	48	S1	48	Located within the the NTCP-binding region of preS1(aa2-48)(10); Identified in previous genotyping studies based on pre-S1(12)
**1374**	S	160	S2	41	Identified in previous study as a key site in determining HBV serotype(13); Work with site-directed mutagenesis confirmed that Lys160 confers serotype *w* reactivity(14)
**1435**	S	221	S	47	Nothing found

Table S4: Top 55 site pairs with highest co-variation (MI>0.6) along the genome (only pairs of sites that include one top 10 site that discriminates genotype). Pairs of sites with very high co-variation are highlighted in orange by MI>0.8.

Top 10 site discriminating genotype	Site presenting high co-variation	Location (gene/domain)	Mutual Information (degree of co-variation)
40	583	Pol/ spacer	0.83
40	1328	S/ pre-S1	0.83
40	1065	Pol/ RT	0.83
40	1432	S/ surface	0.83
40	1403	S/ surface	0.83
40	1411	S/ surface	0.83
40	596	Pol/ spacer	0.82
40	117	HBx	0.77
40	1404	S/ surface	0.76
40	817	Pol/ RT	0.70
40	1311	S/ pre-S1	0.69
40	1406	S/ surface	0.68
40	39	HBx	0.66

Top 10 site discriminating genotype	Site presenting high co-variation	Location (gene/domain)	Mutual Information (degree of co-variation)
40	706	Pol/ spacer	0.64
40	1355	S/ pre-S2	0.64
40	569	Pol/ spacer	0.64
40	539	Pol/ TP	0.60
599	1311	S/ pre-S1	0.89
599	39	HBx	0.89
599	706	Pol/ spacer	0.79
599	1355	S/ pre-S2	0.79
599	583	Pol/ spacer	0.69
599	1432	S/ surface	0.68
599	1411	S/ surface	0.68
599	1065	Pol/ RNAse	0.68
599	1328	S/ pre-S1	0.68
599	1403	S/ surface	0.67
599	596	Pol/ spacer	0.67
599	1371	S/ pre-S2	0.65
599	117	HBx	0.62
599	1404	S/ surface	0.62
599	817	Pol/ RT	0.62
637	542	Pol/ TP	0.74
637	1192	Pol/ TP	0.74
637	1191	Pol/ TP	0.74
637	744	Pol/ RT	0.74
637	543	Pol/ TP	0.74
637	562	Pol/ spacer	0.74
637	610	Pol/ spacer	0.73
637	1259	S/ pre-S1	0.73
637	1212	Pol/ TP	0.72
637	277	C	0.67
637	1211	Pol/ TP	0.67
637	662	Pol/ spacer	0.66
637	434	Pol/ TP	0.66
637	254	C	0.66
637	432	Pol/ TP	0.64
637	615	Pol/ spacer	0.64
637	1264	S/ pre-S2	0.64
637	636	Pol/ spacer	0.64
637	633	Pol/ spacer	0.64
637	1341	S/ pre-S1	0.63
637	571	Pol/ spacer	0.63
637	632	Pol/ spacer	0.62

**Top 10 site discriminating genotype**	**Site presenting high co-variation**	**Location (gene/domain)**	**Mutual Information (degree of co-variation)**
637	466	Pol/ TP	0.61

Supplementary Figures

Figure S1: HBV sequence map for analysis. (top) Layout of HBV genome showing overlapping gene structure. The HBV genome encodes four open reading frames; HBx (X), Core (pre-C, C), Polymerase (P) and Surface (pre-S1, pre-S2, S). (bottom) Concatenated protein sequence generated from the amino acid translations of the HBV genes used for analysis. The gene and domain boundaries are given for Core, Polymerase and Surface, using the gt-A sequence X02763 as a numbering reference.

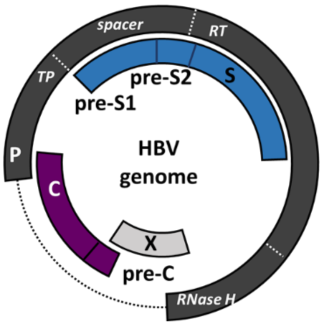

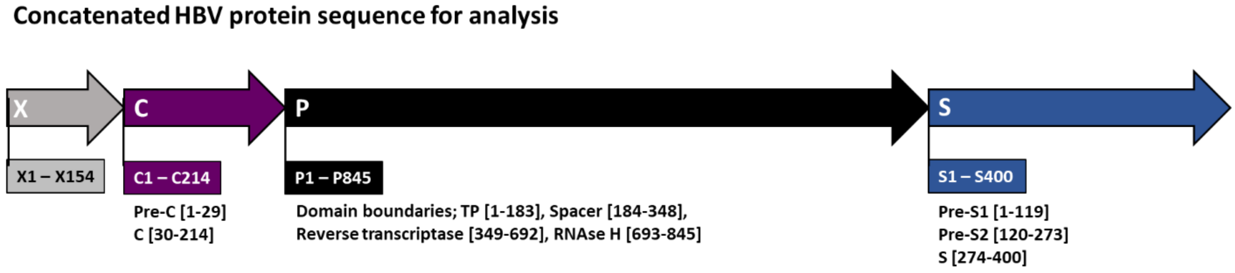

Figure S2: Locations of the top-50 sites within the HBV genome (listed in Table S2).

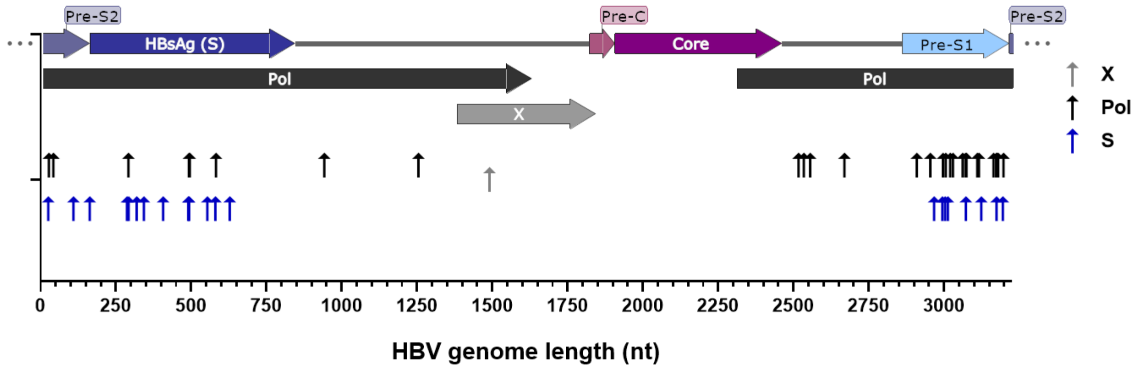

Figure S3: Diagram of the pipeline. Each shape represents a stage or action in the pipeline, and each arrow the flow between stages and actions. The sets of steps described in the main text (1.a-c; 2.a-2b.ii) are identified in the diagram.

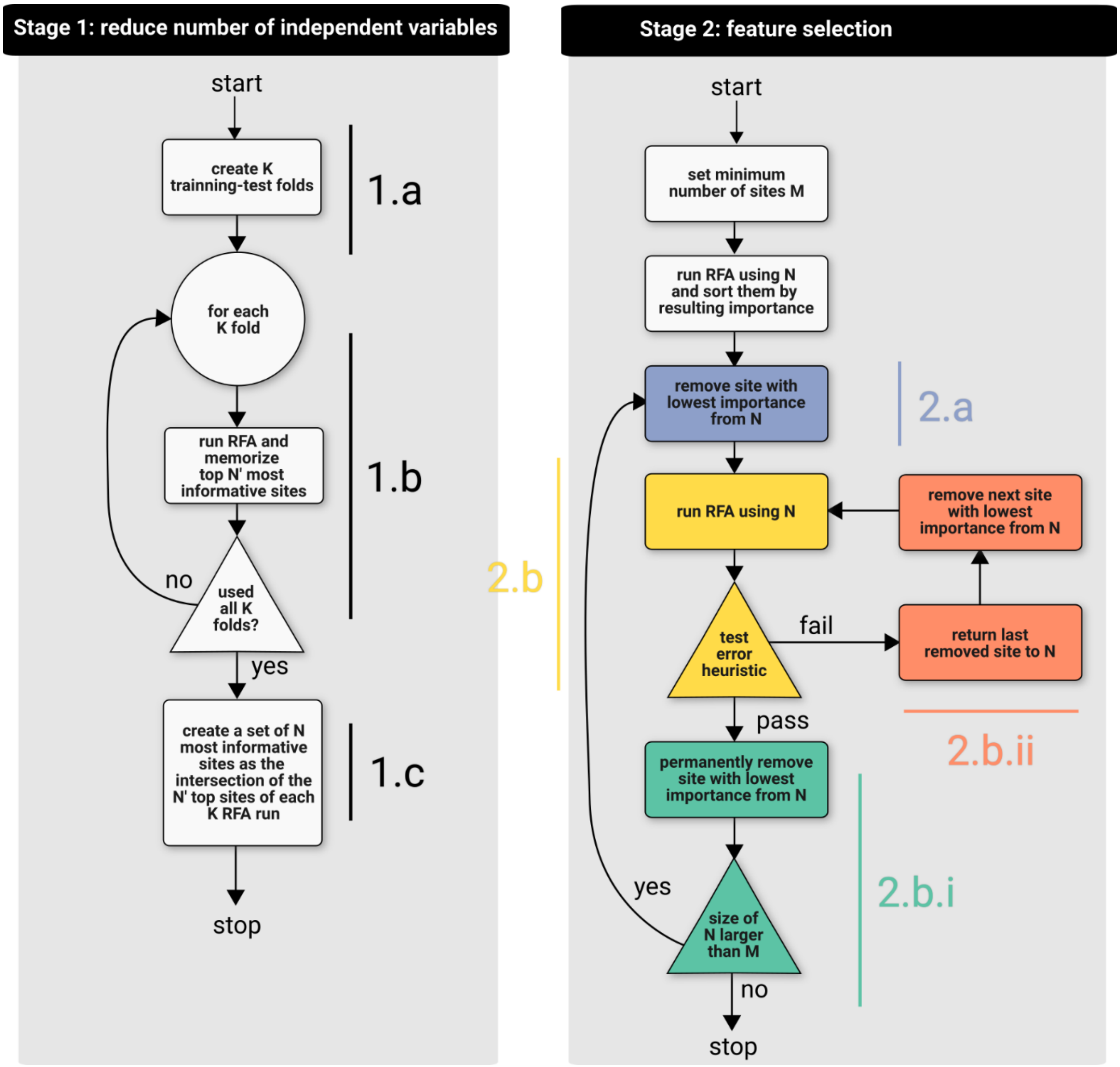

Figure S4: Performance measures of the pipeline during feature selection. Top row: Area under the curve (i.e. multi-class area under the curve, calculated using multiple ROC curves as in (5), accuracy and miss rate given a number of sites (features) used for genotype classification (from left to right, respectively). Bottom row: Same as in top row but restricted to results starting at 40 sites. After N=10 the miss rate (false negative) of genotype F goes above the error heuristic, which is the stop condition for the pipeline. The resulting N=10 sites are the ones described in Tables S2,3.

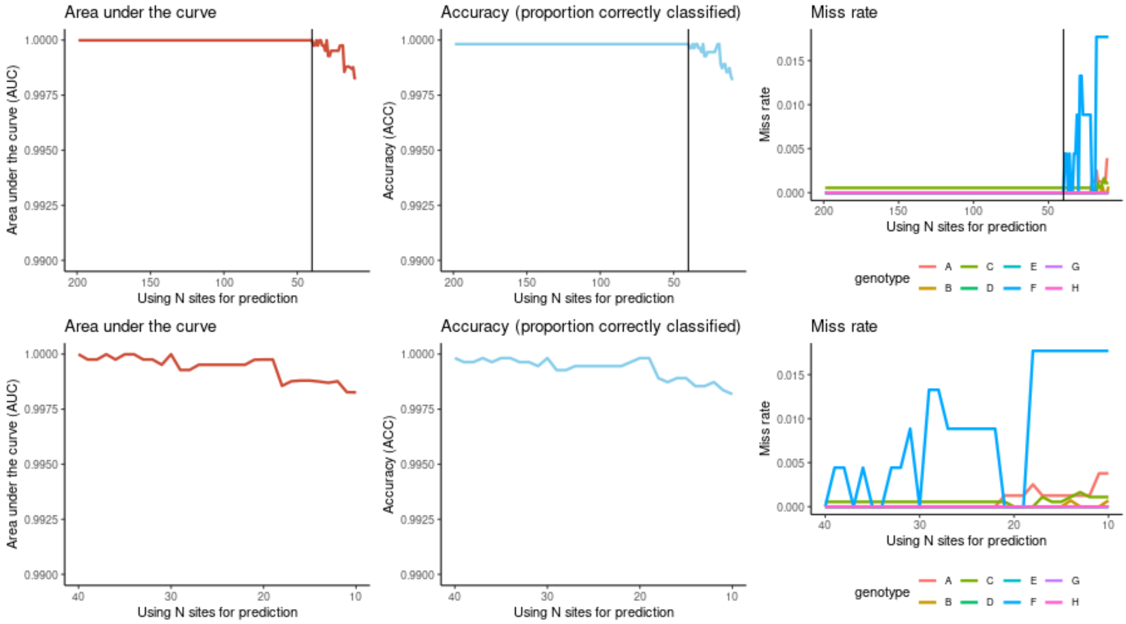

Figure S5: Phylogenetic trees showing (clockwise) the distribution of residues at sites p40 (HBx 40), p570 (Pol 201; spacer), p599 (Pol 230; spacer) and p637 (pol 268; spacer), all mapped by colour. Phylogenetic trees were available to download as a part of the online resource from which we obtained nucleotide sequences(4). Tree nodes where coloured on the basis of the amino acid residue at each site using an inhouse R script based on the R package Analyses of Phylogenetics and Evolution (ape v5.4 (15)). Only the top 5 most frequent amino acids are listed in the color legend, the rest are categorized as “other”. Phylogenies for the other 6 top-10 sites are shown in Figures 2 and S6.

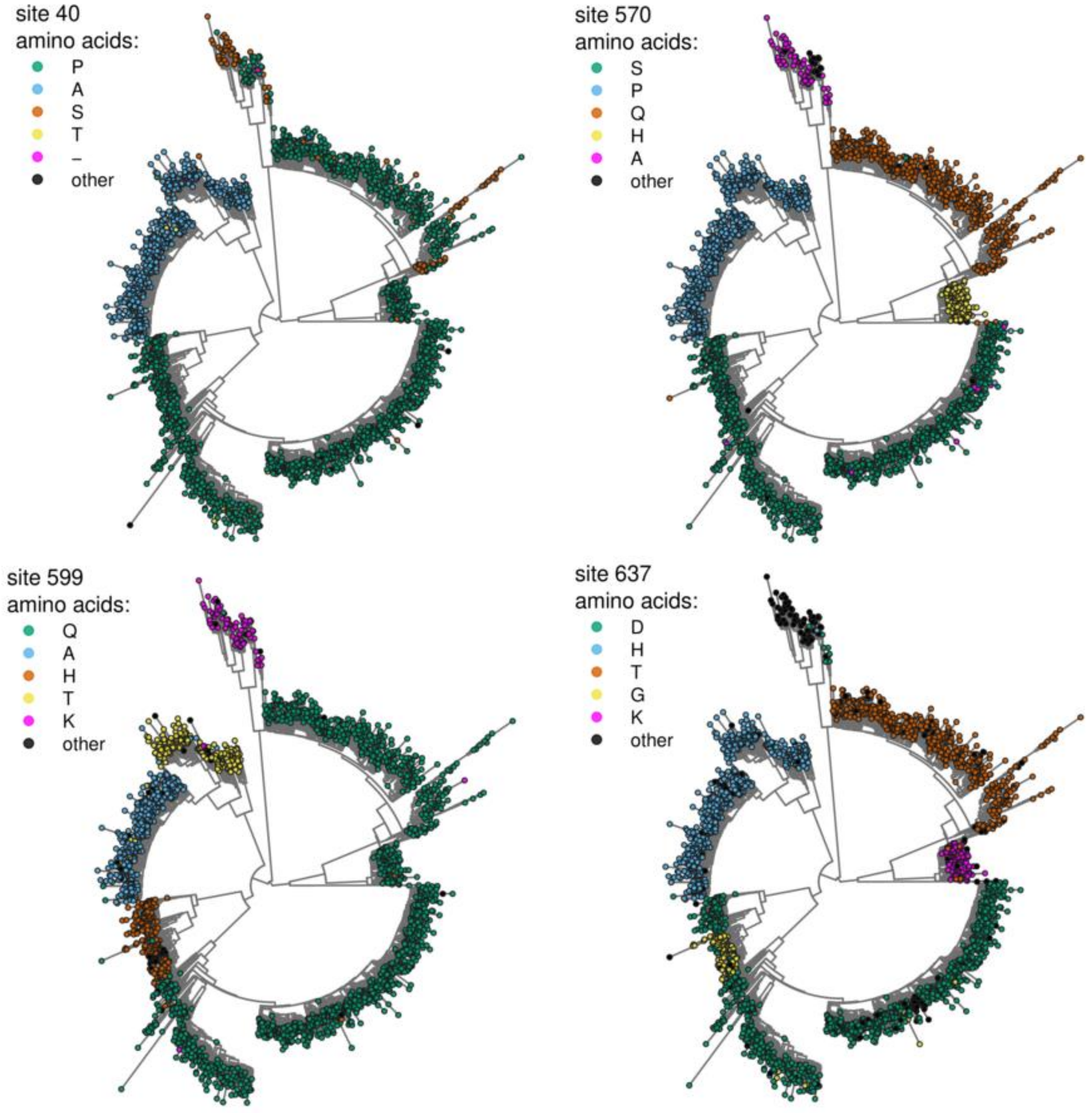

Figure S6: Phylogenetic trees showing (clockwise) the distribution of residues at site p1253 (pre-S1 39), p1262 (pre-S1 48) and p1435 (S 221), all mapped by colour. Phylogenetic trees were available to download as a part of the online resource from which we obtained nucleotide sequences(4). Tree nodes where coloured on the basis of the amino acid residue at each site using an inhouse R script based on the R package 'Analyses of Phylogenetics and Evolution' (ape v5.4 (15)). Only the top 5 most frequent amino acids are listed in the color legend, the rest are categorized as "other". Phylogenies for the other 7 top-10 sites are shown in Figures 2 and S5.

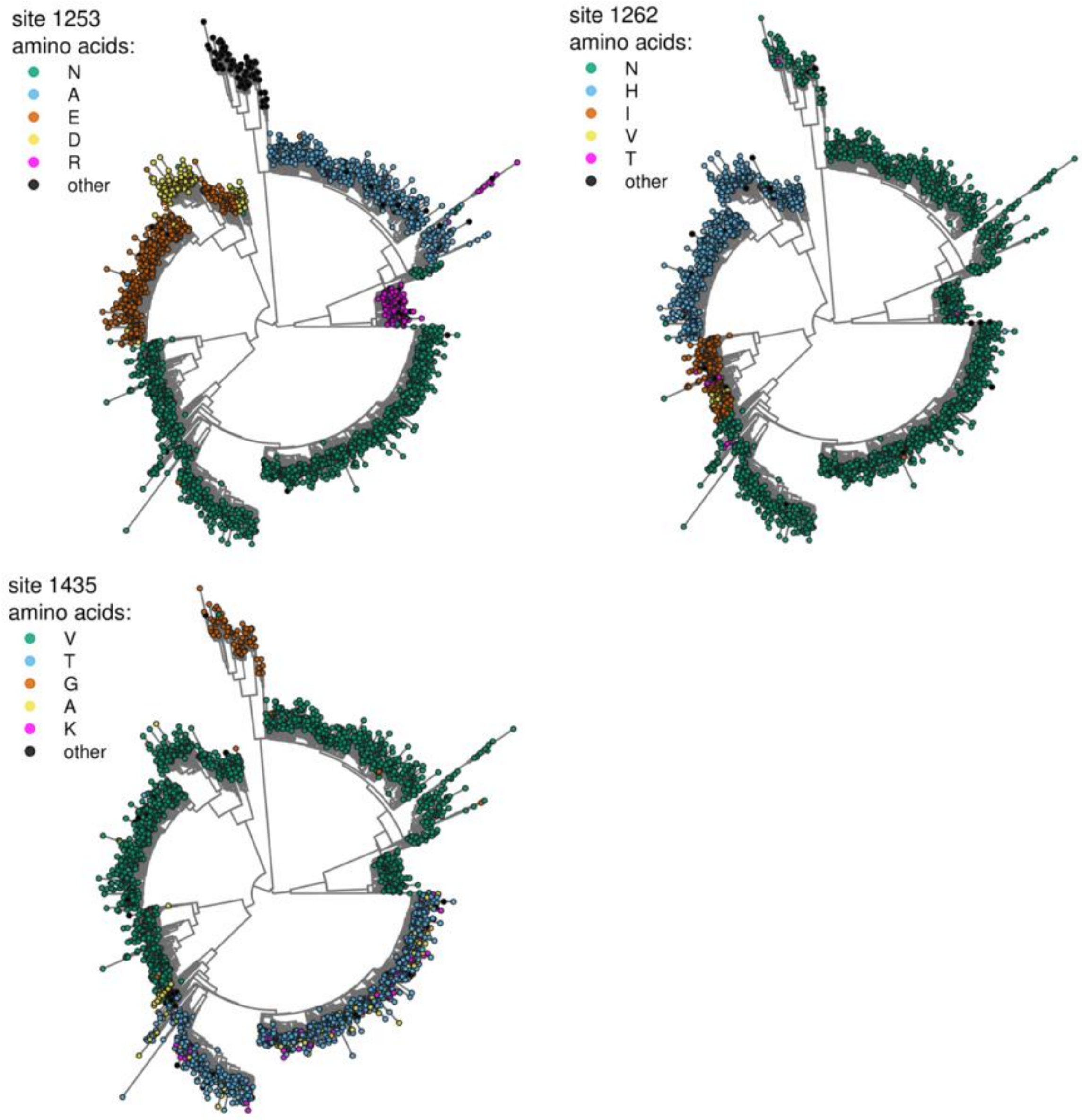

Figure S7: (A) Heatmap of co-variation (MI, color scale) among all possible pairs of sites in the concatenated alignment. Locations of proteins X, C, P and S are presented. (B) Distribution of Mutual Information (MI) among all possible pairs of sites. The red vertical line marks a threshold of intermediate-to-high MI=0.6, and the blue vertical line marks a threshold of very high MI=0.8.. (C-E) Network of sites that show high co-variation with three sites in the top 10 list of sites most informative to predict genotype (center of networks). Link colours represent the level of MI between sites, with presented range of 0.6 – 0.89. The sites presented in panels (C-E) are listed in Table S4 with their respective MI values.

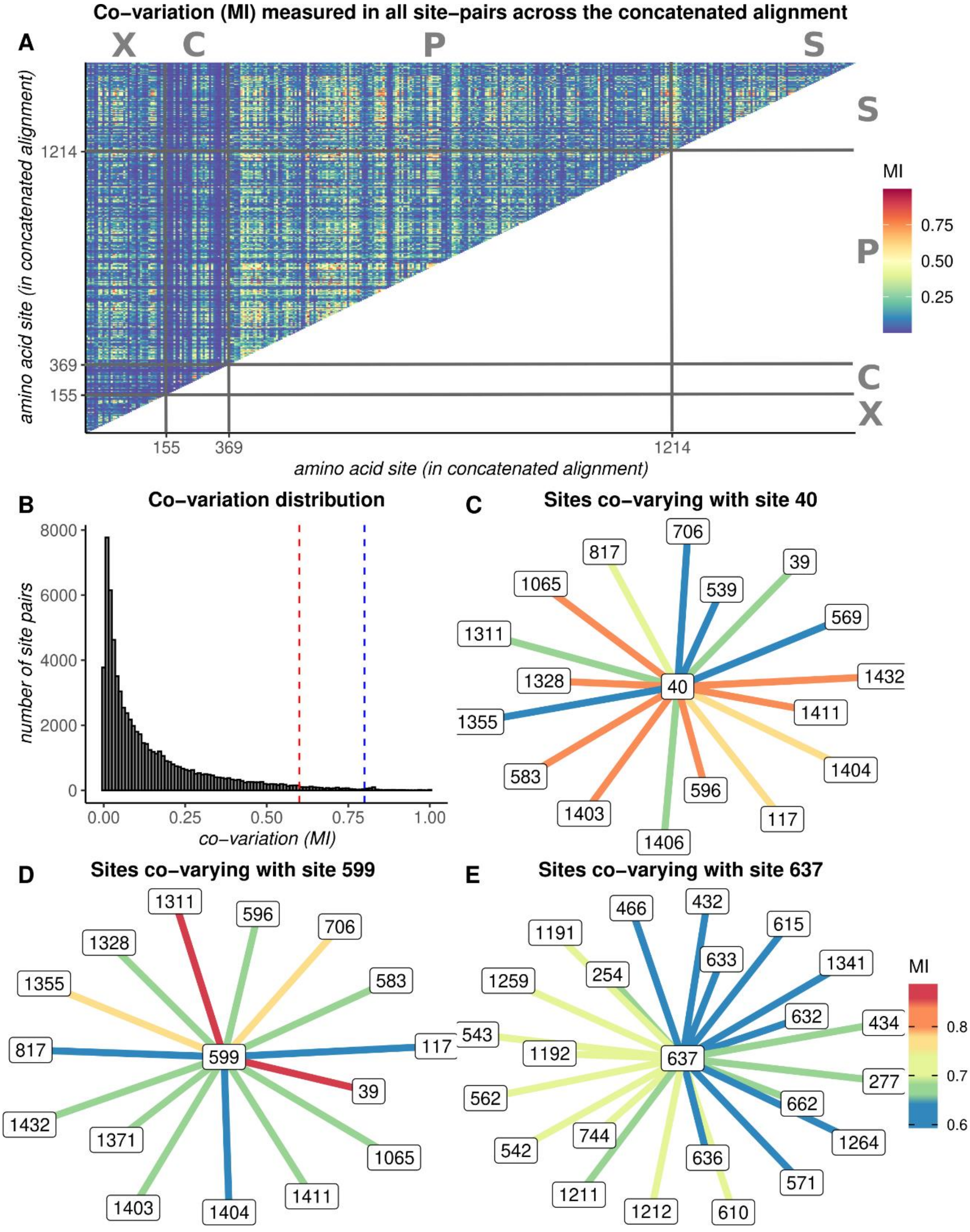

Figure S8: Out-of-bag error distributions for predicting genotypes with sampling the dataset (n=100 independent samples per distribution) using a (A) fixed proportion of samples per genotype, or (B) using a fixed number of samples per genotype. In both panels (A-B), results are presented when prediction is based on all amino acid sites (black) or the top 10 RFA selected sites (grey).

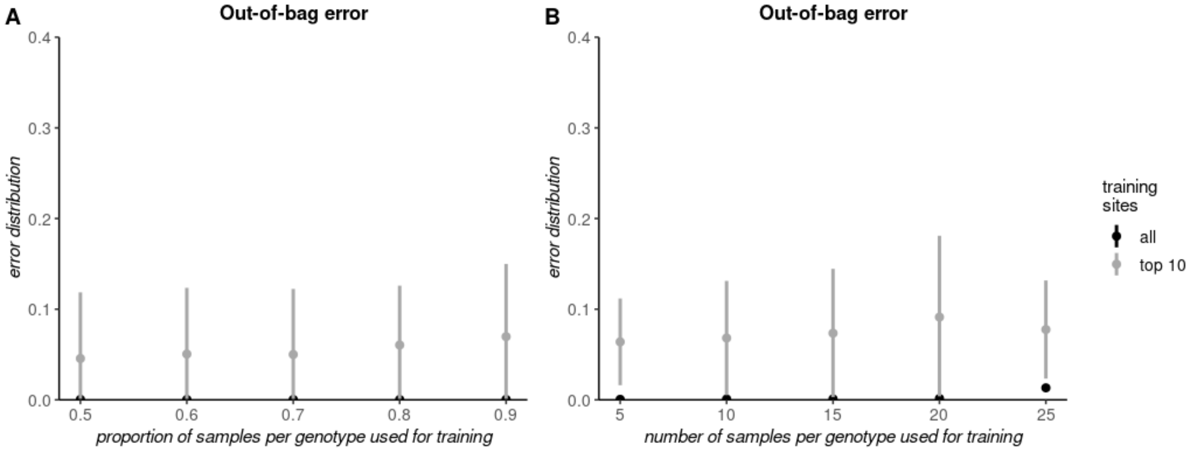

Supplementary References

1. Hayer J, Jadeau F, Deléage G, Kay A, Zoulim F, Combet C. 2013. HBVdb: A knowledge database for Hepatitis B Virus. Nucleic Acids Res 41:566–570.
2. Olinger CM, Jutavijittum P, Hübschen JM, Yousukh A, Samountry B, Thammavong T, Toriyama K, Muller CP. 2008. Possible new hepatitis B virus genotype, southeast Asia. Emerg Infect Dis 14:1777–1780.
3. Tatematsu K, Tanaka Y, Kurbanov F, Sugauchi F, Mano S, Maeshiro T, Nakayoshi T, Wakuta M, Miyakawa Y, Mizokami M. 2009. A genetic variant of hepatitis B virus divergent from known human and ape genotypes isolated from a Japanese patient and provisionally assigned to new genotype J. J Virol 83:10538–10547.
4. Mcnaughton AL, Revill P, Littlejohn M, Matthews PC, Ansari MA. 2020. Analysis of genomic-length HBV sequences to determine genotype and subgenotype reference sequences. J Gen Virol DOI 10.1099/jgv.0.001387.
5. Hand DJ, Robert J Till. 2001. A Simple Generalisation of the Area Under the ROC Curve for Multiple Class Classification Problems. Mach Learn 45:171–186.
6. Spensley KJ, Wikramaratna PS, Penman BS, Walker A, Smith AL, Pybus OG, Jean L, Gupta S, Lourenço J. 2019. Reverse immunodynamics: a new method for identifying targets of protective immunity. Sci Reports 2019 91 9:1–8.
7. Datta S, Banerjee A, Chandra PK, Biswas A, Panigrahi R, Mahapatra PK, Panda CK, Chakrabarti S, Bhattacharya SK, Chakravarty R. 2008. Analysis of hepatitis B virus X gene phylogeny, genetic variability and its impact on pathogenesis: Implications in Eastern Indian HBV carriers. Virology 382:190–198.
8. Lai X, Chen W, Wu Y, Gao Y, Zhang Y, Xu X, Fu Y, Wang X, Yang Y, Zhang Y. 2022. Effect of mutations across reverse transcriptase region on HBV replication and progression of liver diseases in Chinese patients. J Clin Lab Anal 36:1–11.
9. Fu Y, Zeng Y, Chen T, Chen H, Lin N, Lin J, Liu X, Huang E, Wu S, Wu S, Xu S, Wang L, Ou Q. 2019. Characterization and clinical significance of natural variability in hepatitis B virus reverse transcriptase in treatment-naive Chinese patients by Sanger sequencing and next-generation sequencing. J Clin Microbiol 57.
10. Hossain MG, Mahmud MM, Nazmul Hussain Nazir KHM, Ueda K. 2020. PreS1 Mutations Alter the Large HBsAg Antigenicity of a Hepatitis B Virus Strain Isolated in Bangladesh. Int J Mol Sci 21.
11. Chi SW, Maeng CY, Seung JK, Mee SO, Chun JR, Sang JK, Han KH, Hyo JH, Seong ER. 2007. Broadly neutralizing anti-hepatitis B virus antibody reveals a complementarity determining region H3 lid-opening mechanism. Proc Natl Acad Sci U

S A 104:9230.
12. Li S, Wang Z, Li Y, Ding G. 2017. Adaptive evolution of proteins in hepatitis B virus during divergence of genotypes. Sci Reports 2017 71 7:1–11.
13. Purdy MA, Talekar G, Swenson P, Araujo A, Fields H. 2006. A new algorithm for deduction of hepatitis B surface antigen subtype determinants from the amino acid sequence. Intervirology 50:45–51.
14. Hiroaki O, Shigeru O, Yu W, Yukio I, Fumio T, Takeshi T, Yasuhiro A, Yuzo M, Makoto M. 1989. The loss of subtypic determinants in alleles, d/y or w/r, on hepatitis B surface antigen. Mol Immunol 26:197–205.
15. Paradis E, Schliep K. 2019. ape 5.0: an environment for modern phylogenetics and evolutionary analyses in R. Bioinformatics 35:526–528.
